# Simultaneous Application of Red and Blue Light Regulate Carbon and Nitrogen Metabolism, Induces Antioxidant Defense System and Promote Growth in Rice Seedlings under Low Light Stress

**DOI:** 10.3390/ijms241310706

**Published:** 2023-06-27

**Authors:** Maofei Ren, Shanzhen Liu, Guiling Mao, Chengzhu Tang, Panpan Gai, Xiaoli Guo, Huabin Zheng, Weiqin Wang, Qiyuan Tang

**Affiliations:** 1 College of Agronomy, Hunan Agricultural University, Changsha 410128, China; renmaofei2015@163.com (M.R.); 13461625561@163.com (S.L.); 15226333667@163.com (C.T.); 13665514693@163.com (P.G.); hbzheng@hunau.edu.cn (H.Z.); 2 College of Horticulture, Shanxi Agricultural University, Taigu 030801, China; mao1925981573@163.com; 3 College of Agronomy, Henan Agricultural University, Zhengzhou 450046, China; 18838933855@163.com

**Keywords:** rice, red light, blue light, antioxidant defense system, carbon and nitrogen metabolism

## Abstract

The purpose of this study is to determine the effect of light quality on growth, carbon and nitrogen metabolism, and antioxidant defense system of rice seedlings. Six light conditions were employed, including white (W), red (R), blue (B), combined LED of R and B at 3:1 (R3B1), combined LED of R and B at 1:1 (R1B1), as well as combined LED of R and B at 1:3 (R1B3). Combined application of red light and blue light could promote the growth of rice seedling leaves and roots under low light stress to varying degrees, increase the photosynthetic area by increasing the leaf area, improve the root characteristics by increasing the root volume, and increase the dry matter accumulation of rice seedlings. In addition, the combination of red light and blue light could increase carbon and nitrogen metabolites in rice seedling leaves, regulate the expression of genes related to carbon and nitrogen metabolism and enzyme activity, and enhance the antioxidant enzyme activity of rice seedlings. These results indicate that red light and blue light directly have synergistic effects which can regulate the carbon and nitrogen metabolism of rice seedlings, promote the morphogenesis of rice seedlings under low light stress, and promote growth, which has never been reported in previous studies. This study is a new discovery in the application of light quality in crop production and provides new avenues to enhance crop stress resistance. However, further study is needed to explore the physio-biochemical and molecular mechanisms of light quality in crop production.

## 1. Introduction

Rice (*Oryza sativa* L.) is the most important crop in the world and the staple food for more than half of the population. The middle and lower reaches of the Yangtze River are the main rice-producing areas in China. However, in recent years, sunshine duration and total radiation in the middle and lower reaches of the Yangtze River have been decreasing, and the frequency of occurrence of extreme rainy weather has increased significantly, which has a great impact on rice production [1,2]. In particular, early rice in the middle and lower reaches of the Yangtze River was susceptible to low temperature rainy weather such as “late spring coldness”, which resulted in low light stress of rice seedling growth [3]. 

Generally, light is an essential environmental factor for crop growth and has a very important effect on crop growth, yield, and quality [4,5,6]. Low light stress often results in carbon and nitrogen metabolism disorders and disruption of nutrient transport, growth inhibition, and other biochemical and physiological processes. These effects lead to seedling emaciation, low rates of transpiration and photosynthesis, and ultrastructural damage [7,8,9]. This ultimately leads to decreased rice resistance, easy occurrence of disease and insect pests, and decrease yield and quality of the crops [10,11]. It is of great significance for national food security to study the mechanism of low light stress on rice and how to regulate and eliminate the adverse effects of low light stress on rice growth. 

The photoenvironment is an important ecological environmental factors for crop growth and development that mainly affects crop growth and development through photoperiod, optical density, light distribution, and light quality [12,13]. Light quality is an important regulatory factor that affects crop growth and development in the photoenvironment [14]. Light quality is a trigger signal factor which can trigger seed germination, tissue differentiation, and flower bud differentiation [15,16]. Different wavelengths of light carry different levels of energy, which can affect plant growth and development by regulating photoreceptors, transcription factors, and plant hormones [17,18]. Light quality can affect the antioxidant capacity of crops, regulate carbon and nitrogen metabolism, and adapt crops to different abiotic stress environments [19,20]. Red light wavelength is consistent with the peak value of pigment absorption in crop leaves, which can promote cell division and expand cell volume. Blue light can improve photosynthetic capacity per unit leaf area by increasing stomatal opening and quantum yield, and the combination of red and blue light can effectively regulate crop growth. In early rice production in the middle and lower reaches of the Yangtze River, weak seedlings are often caused by low light intensity. Raising seedlings in agricultural facilities requires artificial light to enhance light intensity, and a reasonable combination of effective red light and blue light is necessary to save energy. It is necessary to study the response of rice seedlings to different red and blue light under low light stress. However, little is known about the study of low light stress in plants, especially how light quality enhances low light stress resistance in rice. Therefore, this study aims to investigate the role and mechanism by which light quality regulates rice response to low light stress.

In the present study, two rice varieties, Xiangzaoxian24 (XZX24, conventional indica type rice) and Huazheyou261 (HZY261, hybrid indica type rice), were used as plant materials to study the effects of different light qualities on carbon and nitrogen metabolism and antioxidant defense system under low light stress. The objectives of the present study were to evaluate the influence of different light qualities on the growth of rice seedlings in order to select optimal light proportions of red and blue light to improve seedling quality. The findings not only broaden our understanding of the light-quality-induced low light stress tolerance of rice seedlings, but also shed some light on how light quality regulates the response of rice seedlings to low light stress, thereby improving seedling quality and fostering robust seedlings.

## 2. Results

### 2.1. Morphogenesis

Significant variations in morphogenesis were observed between varieties and light quality treatments (Table 1). The plant height and first leaf sheath length of XZX24 and HZY261 showed a trend of rising with the increase in the red light content. Nevertheless, the stem width showed a downwards trend (Table 1). Plant height and first leaf sheath length of XZX24 and HZY261 all had maximum values in R treatment and were significantly higher than other treatments. Compared with W, significant increases of 27.18% and 25.27% (16.46% and 5.41%) in plant height and first leaf sheath length of XZX24 (HZY261) for R treatment were observed. The stem width of XZX24 and HZY261 all had maximum values in B treatment and was significantly higher than W (R). Compared with W, a significant increase of 29.29% (23.26%) in the stem width of XZX24 (HZY261) for B treatment was observed, respectively. The stem widths of XZX24 and HZY261 were higher than W in some of the treatments (R1B1, R1B3). Nevertheless, the stem widths of XZX24 and HZY261 were higher than R in W treatment. The leaf areas of XZX24 and HZY261 had maximum values in R treatment and were as significantly higher than other treatments. Compared with W, a significant increase of 20.18% (4.12%) in the leaf areas of XZX24 (HZY261) for R treatment was observed. The root volumes of XZX24 and HZY261 showed maximum values in R3B1 treatment and were higher than W. Compared with W, a significant increase of 30.98% (43.69%) in the leaf area of XZX24 (HZY261) for R3B1 treatment was observed. The root growth parameters of XZX24 and HZY261 under combined-light (R3B1, R1B1, R1B3) treatment were significantly higher than that of single-light (R, B) treatment. XZX24 and HZY261 also showed higher root growth parameters in R than B.

### 2.2. Fresh Weight, Dry Weight

**Figure 1:**
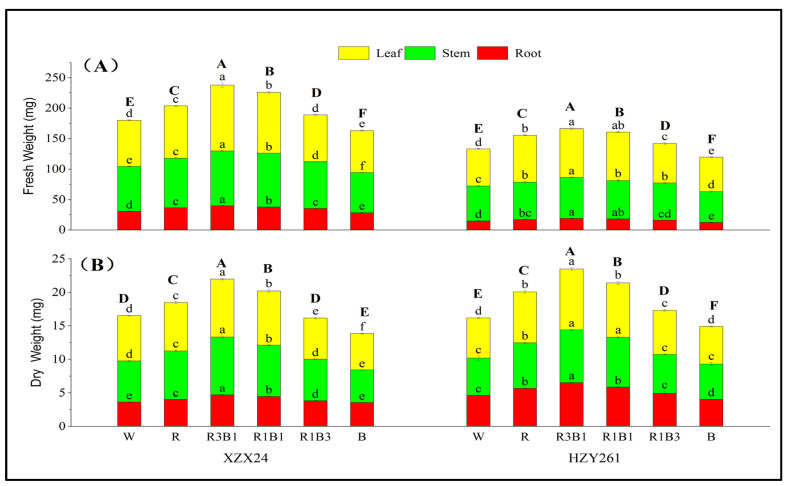
Effects of different light qualities on fresh weight, dry weight of rice seedlings. (**A**): Fresh Weight, (**B**): Dry Weight, XZX24: Xiangzaoxian24, HZY261: Huazheyou261, W: white, R: red, R3B1: 75% red + 25% blue, R1B1: 50% red + 50% blue, R1B3: 25% red + 75% blue, B: blue. Different lowercase letters (capital letters) denote statistical differences among treatments of a cultivar root weight, stem weight and leaf weight (total weight) at the 5% level according to LSD test. Error bars above mean indicate standard error (*n* = 3).

The effects of different light qualities on fresh weight of rice seedlings are summarized in Figure 1A, and the fresh weight of XZX24 was better than HZY261 on the whole. The total fresh weights of XZX24 and HZY261 all had maximum values in R3B1 treatment and were significantly higher than other treatments. Compared with W, a significant increase of 32.04% (24.99%) in the total fresh weight of XZX24 (HZY261) for R3B1 treatment was observed (Figure 1A). The total fresh weight of XZX24 and HZY261 under combined-light (R3B1, R1B1, R1B3) treatment was significantly higher than that of the B treatment. XZX24 and HZY261 also showed higher total fresh weight in R than B.

Significant variations in dry weight were observed between varieties and light quality treatments (Figure 1B). The total dry weight of XZX24 and HZY261 all had maximum values in R3B1 treatment and were higher than in other treatments. Compared with W, a significant increase of 33.03% (45.21%) in the total dry weight of XZX24 (HZY261) for R3B1 treatment was observed (Figure 1B), respectively. The dry weight of XZX24 and HZY261 under combined-light (R3B1, R1B1, R1B3) treatment was higher than that of the B treatment. XZX24 and HZY261 also showed higher dry weight in R than B.

### 2.3. Antioxidant Enzyme Activities, MDA Content

**Figure 2:**
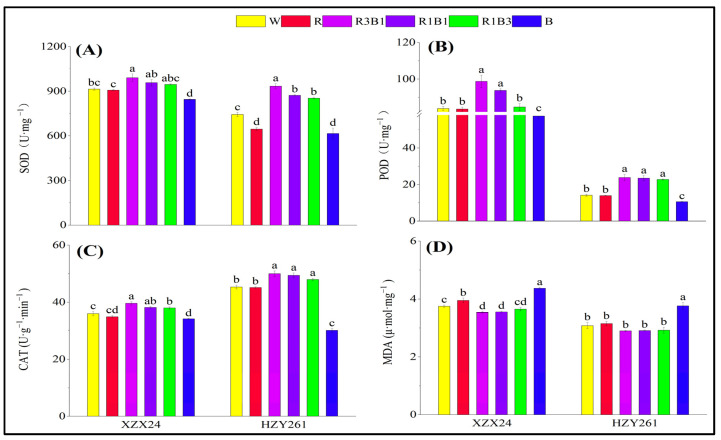
Effects of different light qualities on antioxidant enzyme activities and MDA content of rice seedlings. (**A**): Superoxide Dismutase (SOD), (**B**): Peroxidase (POD), (**C**): Catalase (CAT), (**D**): Malondialdehyde (MDA), XZX24: Xiangzaoxian24, HZY261: Huazheyou261, W: white, R: red, R3B1: 75% red + 25% blue, R1B1: 50% red + 50% blue, R1B3: 25% red + 75% blue, B: blue. Different lowercase letters denote statistical differences among treatments of a cultivar at the 5% level according to LSD test. Error bars above mean indicate standard error (*n* = 3).

Data of the rice seedlings antioxidant enzyme activities (SOD activity, POD activity, CAT activity) along with MDA content are presented in Figure 2. SOD activity, POD activity, and CAT activity of rice seedlings all had maximum values in the R3B1 treatment and were significantly higher than the single-light (R, B) treatment and W treatment. Compared with W, significant increases of 8.29%, 17.71%, and 10.07% (25.65%, 68.33%, and 10.27%), respectively, in SOD activity, POD activity, and CAT activity of XZX24 (HZY261) for R3B1 treatment were observed (Figure 2A–C). In addition, the SOD activity, POD activity, and CAT activity of rice seedlings treated by the combined-light (R3B1, R1B1, R1B3) treatment were higher than that of the single-light (R, B) treatment, and XZX24 and HZY261 also showed higher antioxidant enzyme activities in R than B. The MDA content of rice seedlings all had maximum values in the B treatment and was significantly higher than in other treatments. Compared with W, an increase of 16.59% (22.06%) in MDA content of XZX24 (HZY261) for the B treatment was observed. In addition, the MDA content of rice seedlings treated by the combined-light (R3B1, R1B1, R1B3) treatment was lower than the single-light (R, B) treatment. It can be clearly deduced that a red and blue combination improved seedling antioxidant enzyme activities in the rice plants. At the same time, the combined application of R3B1 treatment was better.

### 2.4. Carbon Metabolite 

**Figure 3:**
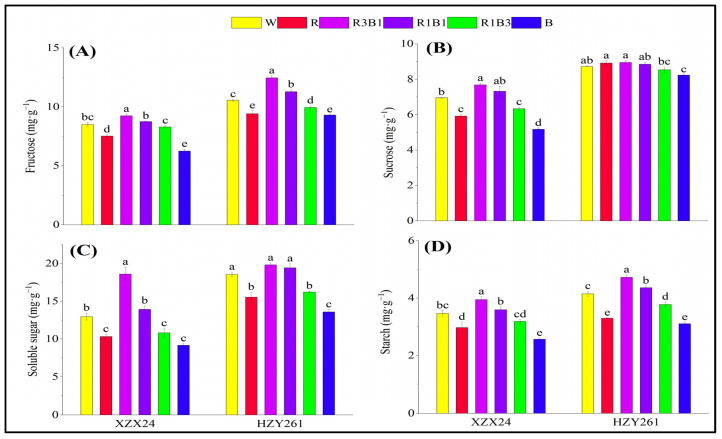
Effects of different light qualities on carbon metabolite of rice seedlings. (**A**): Fructose, (**B**): Sucrose, (**C**): Soluble sugar, (**D**): Starch, XZX24: Xiangzaoxian24, HZY261: Huazheyou261, W: white, R: red, R3B1: 75% red + 25% blue, R1B1: 50% red + 50% blue, R1B3: 25% red + 75% blue, B: blue. Different lowercase letters denote statistical differences among treatments of a cultivar at the 5% level according to LSD test. Error bars above mean indicate standard error (*n* = 3).

The changes in carbon metabolites of the two rice varieties’ (XZX24 and HZY261) rice seedlings under light quality treatments regarding fructose, sucrose, soluble sugar, and starch are shown in Figure 3. The carbon metabolites (fructose, sucrose, soluble sugar, and starch) of rice seedlings all had maximum values in the R3B1 treatment. Compared with W, significant increases of 8.47%, 10.50%, 40.52%, and 13.86% (18.07%, 2.60%, 6.83%, and 13.91%), respectively, in fructose, sucrose, soluble sugar, and starch of XZX24 (HZY261) for R3B1 treatment were observed (Figure 3A–D). In addition, the carbon metabolites (fructose, sucrose, soluble sugar, and starch) of rice seedlings treated by the combined-light (R3B1, R1B1, R1B3) treatment were higher than the single-light (R, B) treatment, except for the sucrose content of HZY261. XZX24 and HZY261 also showed higher carbon metabolites in R than B. Compared with B, increases of 20.32%, 14.51%, 12.68%, and 15.56% (12.90%, 8.38%, 14.36%, and 6.45%), respectively, in fructose, sucrose, soluble sugar, and starch of XZX24 (HZY261) for R treatment were observed.

### 2.5. Soluble Acid Invertase (SAI), Neutral Invertase (NI), Sucrose Synthase (SS), Sucrose Phosphate Synthase (SPS)

**Figure 4:**
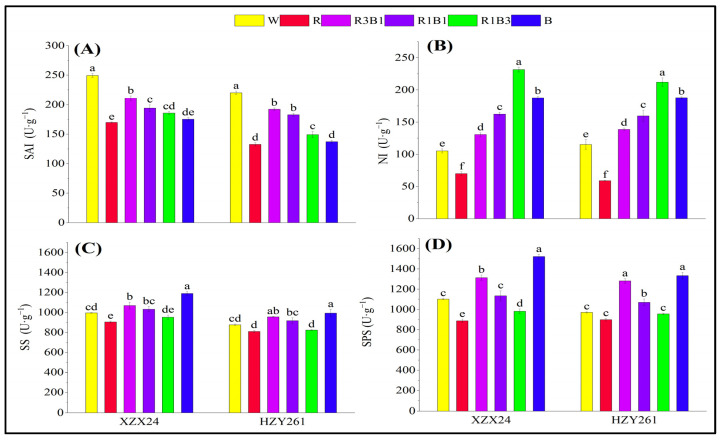
Effects of different light qualities on activities of carbon metabolism enzymes of rice seedlings. (**A**): Soluble acid invertase (SAI), (**B**): Neutral invertase (NI), (**C**): Sucrose synthase (SS), (**D**): Sucrose phosphate synthase (SPS), XZX24: Xiangzaoxian24, HZY261: Huazheyou261, W: white, R: red, R3B1: 75% red + 25% blue, R1B1: 50% red + 50% blue, R1B3: 25% red + 75% blue, B: blue. Different lowercase letters denote statistical differences among treatments of a cultivar at the 5% level according to LSD test. Error bars above mean indicate standard error (*n* = 3).

The SAI of rice seedlings all had maximum values in W treatment (Figure 4A). The SAIs of rice seedlings treated by the combined-light (R3B1, R1B1, R1B3) treatment were higher than the single-light (R, B) treatment, but lower than that of W treatment. XZX24 also showed higher SAI in R than B. Nevertheless, HZY261 also showed higher SAI in B than R, but the differences were not significant. The NI of rice seedlings all had maximum values in R1B3 treatment and was significantly higher than in other treatments. Compared with W treatment, a significant increase of 120.03% (83.87%) in NI of XZX24 (HZY261) for R1B3 treatment was observed (Figure 4B). The NIs of two rice seedling leaves treated with the combined-light (R3B1, R1B1, R1B3) treatment increased with the increase in blue light proportion. In addition, XZX24 and HZY261 also showed higher NIs in B than R.

The SS and SPS of rice seedlings all had maximum values in B treatment (Figure 4C,D). Compared with W, significant increases of 19.51% and 38.11% (13.38% and 37.38%), respectively, in the SS and SPS of XZX24 (HZY261) for B treatment were observed. The SS and SPS of rice seedlings all had maximum values in R3B1 treatment in the combined-light (R3B1, R1B1, R1B3) treatment. Compared with W, significant increases of 7.28% and 19.17% (9.18% and 31.97%), respectively, in the SS and SPS of XZX24 (HZY261) for B treatment were observed. In addition, XZX24 and HZY261 showed higher SS and SPS in B than R, and the difference reached a significant level.

### 2.6. Key Gene Expression Related to Carbon Metabolism 

**Figure 5:**
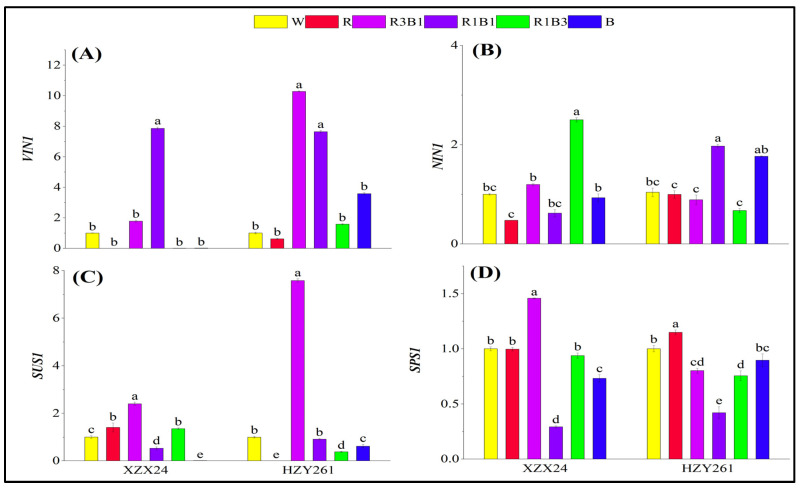
Effects of different light qualities on key gene expression related to carbon metabolism of rice seedlings. (**A**): Relative expression level of *VIN1*, (**B**): Relative expression level of *NIN1*, (**C**): Relative expression level of *SUS1*, (**D**): Relative expression level of *SPS1*, XZX24: Xiangzaoxian24, HZY261: Huazheyou261, W: white, R: red, R3B1: 75% red + 25% blue, R1B1: 50% red + 50% blue, R1B3: 25% red + 75% blue, B: blue. Different lowercase letters denote statistical differences among treatments of a cultivar at the 5% level according to LSD test. Error bars above mean indicate standard error (*n* = 3).

The changes in key gene expression related to carbon metabolism of the two rice varieties (XZX24 and HZY261) rice seedlings under light quality treatments regarding relative expression level of *VIN1*, *NIN1*, *SUS1,* and *SPS1* are shown in Figure 5. Varying degrees of upregulation of *VIN1* expression of XZX24 were observed under the R3B1 and R1B1 treatment relative to the W treatment; varying degrees of upregulation of *VIN1* expression of HZY261 were observed under the R1B1, R1B3, R3B1, and B treatments relative to the W treatment (Figure 5A). In addition, varying degrees of upregulation of *NIN1* expression of XZX24 were observed under the R3B1 and R1B3 treatment relative to the W treatment; varying degrees of upregulation of *NIN1* expression of HZY261 were observed under the R1B1 and B treatment relative to the W treatment (Figure 5B). Varying degrees of upregulation of *SUS1* expression of XZX24 were observed under the R, R3B1, and R1B3 treatment relative to the W treatment (Figure 5C), and the upregulation of *SUS1* expression of HZY261 was observed under the R3B1 treatment. Upregulation of *SPS1* expression of XZX24 was observed under the R3B1 treatment relative to the W (Figure 5D). Nevertheless, upregulation of *SPS1* expression of HZY261 was observed under the R treatment. Relative to W, *VIN1*, and *NIN1* expressions of XZX24 were downregulated by 98.36% and 6.98%, respectively, under single blue light (B) treatment; however, *VIN1* and *NIN1* expressions of HZY261 were upregulated by 257.34% and 69.79%. In addition, *SUS1* and *SPS1* expressions of XZX24 (HZY261) were downregulated by 99.13% and 26.85% (38.00% and 10.42%). Relative to W, *VIN1* and *NIN1* expressions of XZX24 (HZY261) were downregulated by 99.84% and 52.54% (38.56% and 4.17%) under single red light (R) treatment. 

### 2.7. Nitrogen Metabolite 

**Figure 6:**
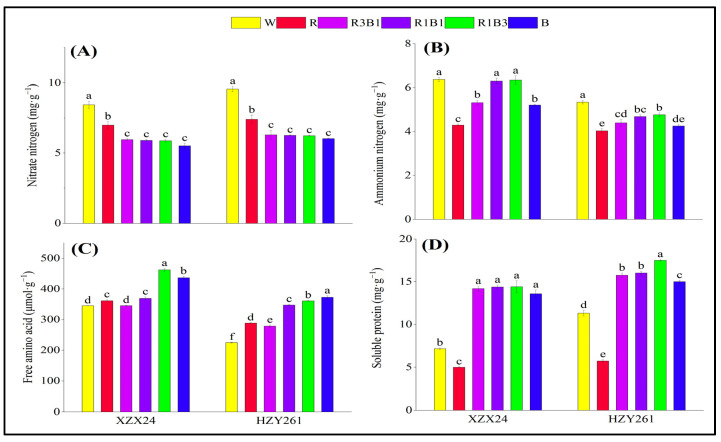
Effects of different light qualities on nitrogen metabolite of rice seedlings. (**A**): Nitrate nitrogen, (**B**): Ammonium nitrogen, (**C**): Free amino acid, (**D**): Soluble protein, XZX24: Xiangzaoxian24, HZY261: Huazheyou261, W: white, R: red, R3B1: 75% red + 25% blue, R1B1: 50% red + 50% blue, R1B3: 25% red + 75% blue, B: blue. Different lowercase letters denote statistical differences among treatments of a cultivar at the 5% level according to LSD test. Error bars above mean indicate standard error (*n* = 3).

The nitrogen metabolite (nitrate nitrogen, ammonium nitrogen, free amino acid, and soluble protein) of XZX24 and HZY261 was focused on in Figure 6, and significant variations in the nitrogen metabolite were observed between varieties and light quality treatments. The nitrate nitrogen and ammonium nitrogen of rice seedlings all had maximum values in the W treatment (Figure 6A,B). Nevertheless, the ammonium nitrogen of rice seedlings all had maximum values in the R1B3 treatment in the combined-light (R3B1, R1B1, R1B3) treatment. The ammonium nitrogen of two rice seedling leaves treated with the combined-light (R3B1, R1B1, R1B3) treatment increased with the increase of blue light proportion. In addition, XZX24 and HZY261 showed higher nitrate nitrogen in R than B, and the difference reached a significant level; however, XZX24 and HZY261 showed higher ammonium nitrogen in B than R.

The free amino acid of XZX24 rice seedlings had maximum values in the R1B3 treatment (Figure 6C); however, HZY261 rice seedlings had maximum values in the B treatment. Compared with W, a significant increase of 33.95% (65.73%) in the free amino acid of XZX24 (HZY261) for R1B3 (B) treatment was observed. In addition, the soluble protein of rice seedlings all had maximum values in R1B3 treatment (Figure 6D). Compared with W, a significant increase of 101.40% (54.46%) in the soluble protein of XZX24 (HZY261) for R1B3 treatment was observed. Nevertheless, the free amino acid and soluble protein of rice seedlings all had maximum values in R1B3 treatment in the combined-light (R3B1, R1B1, R1B3) treatment, and the free amino acid and soluble protein of two rice seedling leaves treated with the combined-light (R3B1, R1B1, R1B3) treatment increased with the increased of blue light proportion. In addition, XZX24 and HZY261 showed higher free amino acid and soluble protein in B than R. Compared with R, significant increases of 26.46% and 172.14% (65.73% and 161.61%) in the free amino acid and soluble protein of XZX24 (HZY261) for B treatment were observed. 

### 2.8. Nitrate Reductase (NR), Asparagine Synthetase (AS), Glutamate Synthetase (GOGAT), Glutamine Synthesis (GS)

**Figure 7:**
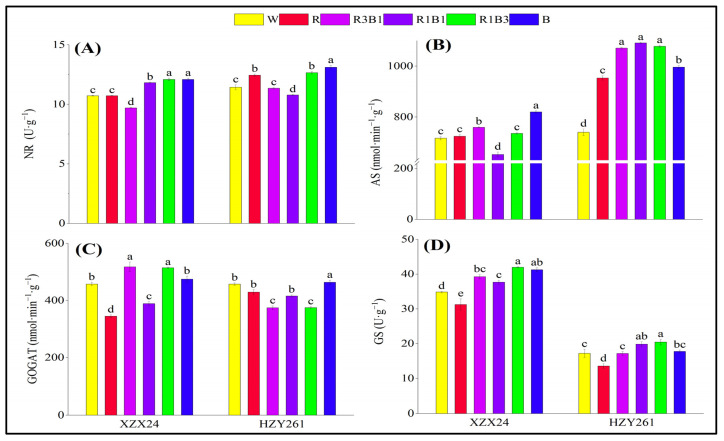
Effects of different light qualities on activities of nitrogen metabolism enzymes of rice seedlings. (**A**): Nitrate reductase (NR), (**B**): Asparagine synthetase (AS), (**C**): Glutamate synthetase (GOGAT), (**D**): Glutamine synthesis (GS), XZX24: Xiangzaoxian24, HZY261: Huazheyou261, W: white, R: red, R3B1: 75% red + 25% blue, R1B1: 50% red + 50% blue, R1B3: 25% red + 75% blue, B: blue. Different lowercase letters denote statistical differences among treatments of a cultivar at the 5% level according to LSD test. Error bars above mean indicate standard error (*n* = 3).

The changes in activities of nitrogen metabolism enzymes of the two rice varieties (XZX24 and HZY261) rice seedlings under light quality treatments regarding nitrate reductase (NR), asparagine synthetase (AS), glutamate synthetase (GOGAT) and glutamine synthesis (GS) are shown in Figure 7. The NR of rice seedlings all had maximum values in the B treatment (Figure 7A). Compared with the W treatment, a significant increase of 12.78% (14.61%) in the NR of XZX24 (HZY261) for the B treatment were observed. The AS of XZX24 rice seedlings had maximum values in the B treatment (Figure 7B); however, HZY261 rice seedlings had maximum values in the R1B1 treatment. Compared with the W treatment, a significant increase of 14.41% (47.40%) in the free amino acid of XZX24 (HZY261) for B (R1B1) treatment was observed. The NR of rice seedlings all had maximum values in R1B3 treatment in the combined-light (R3B1, R1B1, R1B3) treatment, and the NR of two rice seedling leaves treated with the combined-light (R3B1, R1B1, R1B3) treatment increased with the increased of blue light proportion. Nevertheless, the NR of rice seedlings all had maximum values in R3B1 treatment in the combined-light (R3B1, R1B1, R1B3) treatment. However, the AS of XZX24 rice seedlings had maximum values in the R3B1 treatment in the combined-light (R3B1, R1B1, R1B3) treatment; HZY261 rice seedlings had maximum values in the R1B1 treatment. In addition, XZX24 and HZY261 showed higher NR and AS in B than R. Compared with R, significant increases of 12.78% and 13.25% (5.22% and 4.48%) in the NR and AS of XZX24 (HZY261) for B treatment were observed. 

The GOGAT of XZX24 rice seedlings had maximum values in the R3B1 treatment (Figure 7C); however, HZY261 rice seedlings had maximum values in the B treatment. Compared with W, a significant increase of 13.21% (1.42%) in the free amino acid of XZX24 (HZY261) for the R3B1 (B) treatment was observed. The GSs of rice seedlings all had maximum values in the R1B3 treatment (Figure 7D). Compared with the W treatment, a significant increase of 20.37% (18.84%) in the GS of XZX24 (HZY261) for the R1B3 treatment was observed. However, the GOGAT of XZX24 rice seedlings had maximum values in R3B1 treatment in the combined-light (R3B1, R1B1, R1B3) treatment; HZY261 rice seedlings had maximum values in the R1B1 treatment. Nevertheless, the GSs of rice seedlings all had maximum values in the R1B3 treatment in the combined-light (R3B1, R1B1, R1B3) treatment. In addition, XZX24 and HZY261 showed higher GOGAT and GS in B than R. Compared with R, significant increases of 37.52% and 31.96% (8.16% and 31.14%) in the GOGAT and GS of XZX24 (HZY261) for B treatment were observed.

### 2.9. Key Gene Expression Related to Nitrogen Metabolism 

**Figure 8:**
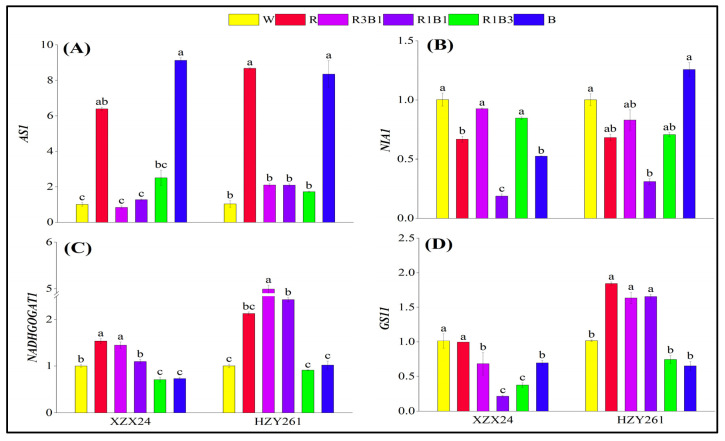
Effects of different light qualities on key gene expression related to nitrogen metabolism of rice seedlings. (**A**): Relative expression level of *AS1*, (**B**): Relative expression level of *NIA1*, (**C**): Relative expression level of *NADHGOGAT1*, (**D**): Relative expression level of *GS11*, XZX24: Xiangzaoxian24, HZY261: Huazheyou261, W: white, R: red, R3B1: 75% red + 25% blue, R1B1: 50% red + 50% blue, R1B3: 25% red + 75% blue, B: blue. Different lowercase letters denote statistical differences among treatments of a cultivar at the 5% level according to LSD test. Error bars above mean indicate standard error (*n* = 3).

The changes in key gene expression related to nitrogen metabolism of the two rice varieties (XZX24 and HZY261) rice seedlings under light quality treatments regarding relative expression levels of *AS1*, *NIA1*, *NADHGOGAT1*, and *GS11* are shown in Figure 8. Herein, varying degrees of upregulation of *AS1* expression of XZX24 and HZY261 were observed under the light quality treatments relative to the W (Figure 8A) treatment, except for the R3B1 treatment of XZX24. Nevertheless, varying degrees of downregulation of *NIA1* expression of XZX24 and HZY261 were observed under the light quality treatments relative to the W (Figure 8B) treatment, except for the B treatment of HZY261. Varying degrees of upregulation of *NADHGOGAT1* expression of XZX24 and HZY261 were observed under the R, R3B1, and R1B1 treatments relative to the W treatment (Figure 8C). Varying degrees of downregulation of *GS11* expression of XZX24 were observed under the light quality treatments relative to the W treatment (Figure 8D). Nevertheless, varying degrees upregulation of *GS11* expression of HZY261 were observed under the R, R3B1, and R1B1 treatments relative to the W treatment. Relative to the W treatment, *AS1* and *NADHGOGAT1* expression of XZX24 (HZY261) were upregulated by 532.93% and 53.21% (738.75% and 112.58%), respectively, under the single red light (R) treatment. Relative to W, *AS1* expression of XZX24 (HZY261) was upregulated by 804.04% (707.46%) under the single blue light (B) treatment; however, *GS11* expression of XZX24 (HZY261) was downregulated by 31.31% (35.90%).

### 2.10. Heat Map Analysis 

**Figure 9:**
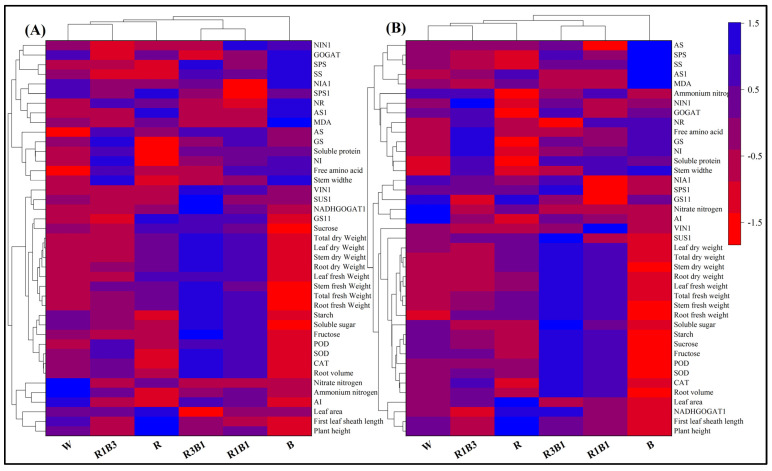
Cluster heat map analysis summarizing rice seedlings responses to light quality treatments in two varieties. (**A**): XZX24 (Xiangzaoxian24), (**B**): HZY261 (Huazheyou261), W: white, R: red, R3B1: 75% red + 25% blue, R1B1: 50% red + 50% blue, R1B3: 25% red + 75% blue, B: blue. Results are visualized using a false color scale with blue indicating an increase and red a decrease of the response parameters.

A heat map synthesizing the response of the measured parameters provided an integrated view of the effect of light quality on the photomorphogenesis, carbon, and nitrogen metabolism of rice seedlings (Figure 9). Most of the measured parameters of XZX24 and HZY261 under R3B1 treatment were significantly higher than of single-light (R, B) treatment. In addition, monochromatic red light and monochromatic blue light treatments showed opposite responses differing in most of the measured parameters.

Among the XZX24 variety, the W and R1B3 clusters are the closest to each other in terms of measured parameter responses, and the R3B1 and R1B1 clusters are the closest to each other in terms of measured parameter responses (Figure 9A). In addition, the W and R1B3 clusters are equidistant from cluster R, and the W, R1B3, and R clusters are equidistant from clusters R3B1 and R1B1. At the same time, cluster R is considerably separated from the other two clusters (W, R1B3): red light reduced SS, SPS, GS, soluble protein, NI, starch, SOD, CAT, ammonium nitrogen, and AI and increased gene (*SPS1*, *AS1*, *GS11*) expression, leaf fresh weight, leaf area, plant height, and first leaf sheath length, contributing to separate the R cluster from the others. Furthermore, cluster B has the furthest distance from clusters W, R1B3, R3B1, R1B1, and R. Cluster B is considerably separated from the other five clusters: blue light reduced gene (*GS11*) expression, sucrose, dry weight (total, root, stem, leaf), fresh weight (total, root, stem, leaf), starch, soluble sugar, fructose, CAT, POD, SOD, root volume, AI, plant height, and first leaf sheath length, and increased gene (*NIA1*, *NIN1*, *AS1*) expression, GOGAT, SS, SPS, NR, MDA and stem width compared to other five treatments, contributing to separate the B cluster from the others.

Among the HZY261 variety, the W and R1B3 clusters are the closest to each other in terms of measured parameter responses, and the R3B1 and R1B1 clusters are the closest to each other in terms of measured parameter responses (Figure 9B). In addition, the W and R1B3 clusters are equidistant from cluster R, and the W, R1B3, and R clusters are equidistant from cluster R3B1 (R1B1). At the same time, cluster R is considerably separated from the other two clusters (W, R1B3): red light reduced gene (*NIN1*) expression, SS, SPS, ammonium nitrogen, GOGAT, GS, NI, soluble protein, stem width, AI, and CAT, and increased gene (*AS1*, *GS11*, *NADHGOGAT1*) expression, leaf area, plant height, and first leaf sheath length, contributing to separate the R cluster from the others. Furthermore, clusters B have the furthest distance from cluster W, R1B3, R3B1, R1B1, and R, and cluster B is considerably separated from the other five clusters: blue light reduced gene (*SUS1*, *NADHGOGAT1*) expression, dry weight (total, root, stem, leaf), fresh weight (total, root, stem, leaf), soluble sugar, starch, sucrose, fructose, CAT, POD, SOD, root volume, leaf area, plant height, and first leaf sheath length, and increased gene (*AS1*) expression, AS, SS, SPS, MDA, NR, free amino acid, GS, NI, and stem width, compared to other five treatments, contributing to separate the B cluster from the others.

## 3. Discussion

### 3.1. Red and Blue Light Can Regulate the Early Morphogenesis in Rice

Light quality is one of the most important light factors in a crop growth environment. Light quality plays an important role in regulating crop seed germination, cell growth and differentiation, photosynthesis, and carbon and nitrogen metabolism. Therefore, the external morphological characteristics of crops are the most intuitive manifestation of their adaptability to the light environment. In this study, the effects of different light quality treatments on seedling growth of two rice varieties (XZX24 and HZY261) were investigated. The results of the present study are consistent with the previous research [14,21] and report that the plant height, first leaf sheath length, leaf area, and root volume of XZX24 and HZY261 under the R treatment were higher than those under the B treatment (Table 1); these indexes of XZX24 and HZY261 were significantly weaker than those of the monochrome R treatment. Compared with previous research results [14,21], although the degree of promoting cell elongation is different, it can confirm that red light can promote the longitudinal development of crop cells. However, the stem width of XZX24 and HZY261 under B treatment were higher than those under R treatment (Table 1) [22,23]. Meanwhile, the fresh weight and dry weight of XZX24 and HZY261 under R treatment were also greater than those under B treatment (Figure 1). This indicated that red light could promote the elongation growth and dry matter accumulation of rice seedlings, which was consistent with results in Gossypium hirutum [24] and Chrysanthemum morifolium [25]. The differences between research might be attributed to different spectrum absorption range in different plant materials and varieties and other factors including temperature, irrigate, O_2_ content, CO_2_ content, relative air humidity, and fertilizer and crop management. The effects of comprehensive environmental factors on the morphogenesis characteristics of rice seedlings need to be further studied.

Compared with single-light, combined-light is more conducive to plant morphogenesis [26,27]. When approximately 25% of the red light in R treatment was replaced by blue light, the fresh weight, dry weight, and root volume of XZX24 and HZY261 all increased statistically in R3B1 treatment (Table 1, Figure 1). When approximately 75% of the red light in the R treatment was replaced by blue light, the plant height, first leaf sheath length, leaf area, fresh weight, and dry weight of XZX24 and HZY261 all decreased statistically in the R1B3 treatment (Table 1, Figure 1). Our experimental results showed that an appropriate proportion of blue light was helpful to promote the main effect of red light. Nevertheless, an excessive proportion of blue light counteracts the function of red light. Previous studies has reported that red light promotes stem elongation in tomatoes; this effect can be reversed by adding a certain proportion of blue light [28,29]. This result also revealed the complexity of light quality regulation in plant morphogenesis [30,31]. For rice seedlings, leaves are an important part of photosynthesis; roots absorb nutrients and water; and plant height and stem width are related to the stress resistance of the plant. Generally speaking, in the case of the same stem width, the higher the plant height, the higher the plant stress resistance may be; similarly, in the case of the same plant height, the larger the stem width, the higher the plant stress resistance may be. This also indicates that our experiment provides a suitable light quality value ratio for rice seedling growth. In addition, the values of morphogenesis, fresh weight, and dry weight of XZX24 were higher than HZY261, which showed that XZX24 has better light quality adaptability than HZY261.

### 3.2. Red and Blue Light Can Regulate Carbon Metabolism of Rice Seedlings

Carbohydrates are important organic metabolites in plants. As the products of plant photosynthesis, carbohydrates are the main energy substances to maintain plant life activities. Photosynthetic pigments in plant leaves can absorb, transfer, and transform light energy, which is the basis of plant photosynthesis. The composition and content of photosynthetic pigments have an important effect on leaf photosynthesis, and light quality will affect the formation and accumulation of photosynthetic pigments in plants [32,33]. The photosynthesis process is divided into light reaction and dark reaction, in which the light reaction stage is mainly composed of photosystem I (PSI) and photosynthetic system II (PSII). PSI is before PSII, but electron transfer first starts from PSII. Light quality affects the activity of PSII and thus affects plant photosynthesis [34,35]. Hamdani et al. [36] found that blue light induction significantly reduced Fv/Fm of rice photosynthetic performance, and the larger the initial rate of NPQ induction, the higher the NPQ, corresponding to the qE component of NPQ. The lower the maximum mass subyield of PSII, however, under long-term red light treatment of rice, decreased PSII and increased NPQ; Fv/Fm did not change. In this study, the effects of light quality on photosynthetic products (carbohydrates) of rice seedlings were introduced. Further studies on photosynthetic pigments, light receptors, and functional activity of the photosynthetic apparatus are still needed.

The higher the content of carbohydrates, the more beneficial it is to the growth of plants. The level of carbohydrate content can be used as one of the main markers of carbon metabolism in plants. Light quality affects the accumulation of photosynthetic products (carbohydrates) and the activities of carbon metabolizing enzymes (SAI, NI, SS, SPS) [37,38,39]. The results of the present study were that the fructose, sucrose, soluble sugar, and starch of XZX24 and HZY261 under the R treatment were higher than those under the B treatment (Figure 3) [40,41]. At the same time, it was found in the study that 25% blue light replacement of red light resulted in higher carbohydrate content than other treatments, indicating that the addition of blue light on the basis of red light was beneficial to carbohydrate accumulation [42]. In order to regulate the production, transport, and accumulation of carbohydrates, crops need a reasonable proportion of red light and blue light during the growth process. In addition, the combined treatment of red light and blue light also improved enzyme activity related to carbon metabolism and the expression of key genes to varying degrees (Figure 3 and Figure 4). The combination of red light and blue light can regulate the expression of genes related to carbon metabolism and further improve or reduce the activity of carbon metabolism enzymes to regulate the process of carbon metabolism [43,44]. However, the regulation of carbon metabolism gene expression and carbon metabolism enzyme activity by the combination of red and blue light is caused by the complex physiological mechanism of plant carbon metabolism, rather than simple additive effects. In this study, the expression of some genes differs greatly among different rice varieties: The expression of SUS1 in R3B1-treated HZY261 is exceptionally high compared to XZX24 and other light quality treated HZ261, which may be the reason of the difference of photoreceptors in different rice varieties, which still needs to be further studied.

### 3.3. Red and Blue Light Can Regulate Nitrogen Metabolism of Rice Seedlings

Nitrogen metabolism is an important physiological activity of plant growth and development. Plants absorb nitrogen through nitrogen assimilation and provide nitrogen for growth and development. Plants mainly absorb and utilize nitrogen in soil (mainly nitrate nitrogen and ammonium nitrogen) through roots, so the development of roots directly affects plant nitrogen metabolism, and light quality can indirectly regulate plant nitrogen metabolism by affecting root growth [37]. At the same time, light quality will also directly participate in other aspects of plant nitrogen metabolism, affecting the content of physiological activities related to nitrogen metabolism (nitrate nitrogen, ammonium nitrogen, free amino acid, and soluble protein) and the activities of related nitrogen metabolism enzymes (NR, AS, GOGAT, GS) [45,46]. The results of the present study are that the nitrate nitrogen of XZX24 and HZY261 under the R treatment was higher than those under the B treatment (Figure 6A); nevertheless, ammonium nitrogen, free amino acid, and soluble protein of XZX24 and HZY261 under the B treatment were higher than those under the R treatment (Figure 6B–D). As the ratio of red and blue light changes, so do nitrogen metabolites. The content of ammonium nitrogen, free amino acid, and soluble protein in rice seedling was increased by increasing the proportion of blue light. In addition, the combined treatment of red light and blue light also improved the enzyme activity related to nitrogen metabolism and the expression of key genes (*NIN1*) to varying degrees (Figure 7 and Figure 8). The combination of red light and blue light can regulate the expression of genes related to nitrogen metabolism and further improve or reduce the activity of nitrogen metabolism enzymes to regulate the process of nitrogen metabolism [47,48].

Physiological changes of plant carbon and nitrogen metabolism can regulate the photosynthesis of plant leaves [49] and affect the absorption of mineral elements by plant roots and the synthesis of related proteins and enzymes. Nitrogen metabolism depends on carbon metabolism to provide energy and carbon source. At the same time, nitrogen metabolism provides photosynthetic pigments and related enzymes necessary for carbon metabolism. These two metabolic activities require common reducing power and the carbon skeleton and carbon and nitrogen metabolism coordinate, promote each other, and have a close relationship. Jointly, plant growth and development is regulated. However, the molecular mechanism of the combination of red light and blue light to further regulate crop growth by regulating carbon and nitrogen metabolism remains to be further studied.

### 3.4. The Combination of Red Light and Blue Light Can Improve the Low Light Tolerance of Rice Seedlings

The light environment is one of the important ecological environmental factors for plant growth and development. It mainly affects plant growth and development through photoperiod, light density, light distribution, and light quality. Under low light stress, the rice plant height increased, the stem base width decreased, the leaf thinned, photosynthetic pigment content decreased, and net photosynthetic rate decreased; this disrupted the accumulation, transport, and distribution of photosynthetic products and led to the decline of rice low light resistance. Studies have shown that light quality can regulate plant carbon and nitrogen metabolism and enhance plant low light resistance. In this study, it was found that light quality can affect the low light resistance of rice, and the combination of red and blue light can significantly improve the antioxidant enzyme activity of rice seedlings (Figure 2), reduce the content of MDA, and eliminate more reactive oxygen species. Under low light stress, the antioxidant system of different rice varieties treated with light quality is different. For example, the POD content of XZX24 and HZY261 was found to be very different in the study (Figure 2B), which may be caused by the different adaptability of different rice varieties to low light. The combined treatment of red and blue light significantly increased the content of soluble solids (soluble sugar and soluble protein) in rice seedlings (Figure 3 and Figure 6) and enhanced the low light resistance of rice seedlings. We found that the combination of red and blue light can regulate the physiological metabolism of rice and enhance the resistance to low light by regulating the expression of genes related to carbon and nitrogen metabolism under low light (Figure 5 and Figure 8), which has never been reported in previous studies. Nevertheless, further study are needed to explore the physio-biochemical and molecular mechanisms of red–blue light combination in crop production in enhancing low light resistance.

## 4. Materials and Methods

### 4.1. Plant Materials and Description of Experiment

The seeds of two rice varieties (XZX24 and HZY261) used in this study were collected from Hunan Jinse Nongfeng Seed Industry Co., Ltd., Changsha, China and Hunan Jinjian Seed Industry Technology Co., Ltd., Changsha, China. Experiments were conducted in an artificial climate chamber at Hunan Agriculture University, Changsha, China. The germinated rice (XZX24 and HZY261) seeds (*n* = 3 per hole) were sown in 106-hole (Bottom diameter/calibre/height: 10 mm/17 mm/20 mm) seedling trays with nutrient soil (Hunan Xianghui Agricultural Technology Development Co., Ltd., Shaoyang, China) and placed in an artificial climate chamber (YHMR-1000, Ningbo Yanghui Instrument Co., Ltd., Ningbo, China).

**Figure 10:**
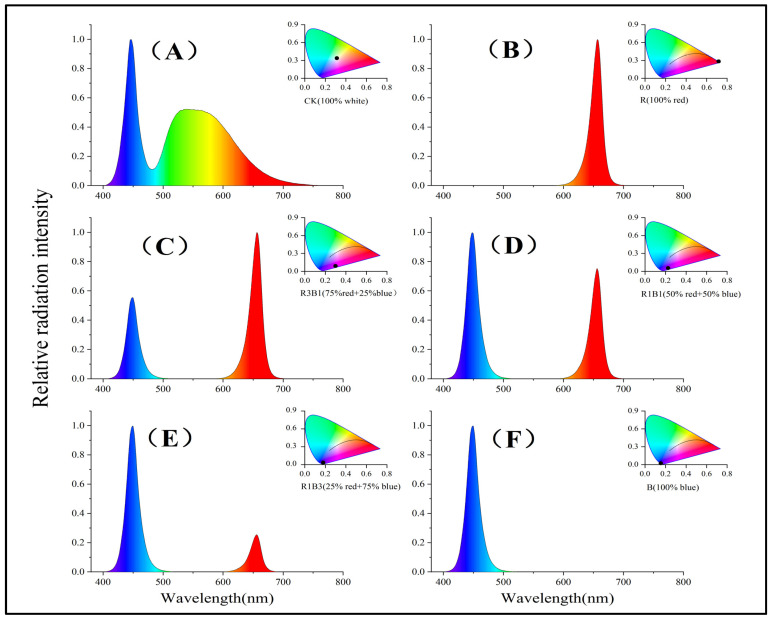
Relative spectral value of six treatments. (**A**): W, 100% white, (**B**): R, 100% red, (**C**): R3B1, 75% red + 25% blue, (**D**): R1B1, 50% red + 50% blue, (**E**): R1B3, 25% red + 75% blue, (**F**): B, 100% blue.

When the rice seedlings’ first true leaves were expanded and after 1 day of preculture under white LED illumination with 12 h of photoperiod (100 μmol m^−2^ s^−1^), 25 °C/15 °C (daytime/night), and 70%/80% (daytime/night) relative humidity, continued culture was carried out under different light quality conditions. The seedlings were subjected to six experimental illumination treatments. The six light treatments growth conditions are as follows: (1) W, white light; (2) R, red light; (3) R3B1, 75% red + 25% blue light; (4) R1B1, 50% red + 50% blue light; (5) R1B3, 25% red + 75% blue light; (6) B, blue light, and average relative humidity = 70%/80% (daytime/night), temperature = 25 °C/15 °C (day/night), light intensity = 100 μmol m^−2^ s^−1^, and photoperiod = 12 h/12 h (day/night). Photon fluxes were measured with a spectroradiometer (PLA-30, Everfine Optoelectronic Information Co., Ltd., Hangzhou, China). The light intensity was adjusted on the basis of the distance between the light source and the rice seedlings canopy. The center wavelength of each light was as follows: red light, 665 nm; blue light, 450 nm. The spectral values of the four treatments are shown in Figure 10.

The rice seedlings were organized in a complete randomized block design with four replicates per treatment. After 21 days of cultivation, the rice seedlings plants under different treatments were sampled, and the related indexes were determined. The sample parts were immediately frozen in liquid nitrogen and stored at −80 °C until analysis.

### 4.2. Morphological Indicators

Twenty-one days after treatment, the rice seedlings were harvested. The plant height, first leaf sheath length, and stem width were examined using a rectilinear scale (vernier caliper). Five rice seedlings were selected for each replicate, and the root system and the leaf were analyzed by scanner (Epson Perfection V850 Pro, Epson China Co., Ltd., Shanghai, China) and LA-S analyzer system (LA-S, Hangzhou Wanshen Testing Technology Co., Ltd., Hangzhou, China) to obtain the related data of root volume and leaf area. The plants, root, stem, leaf fresh weight, and dry weight were examined using an electronic balance. Fifty plants were then randomly selected from each replicate; for dry weight, they were deactivated at 110 °C and then dried at 70 °C for 96 h.

### 4.3. Antioxidant Enzyme Activities and MDA Content

Extracts of Antioxidant Enzyme Activity: To prepare the tissue homogenate, 0.5 g of rice seedlings leaves was accurately weighed and ground in 5 mL of distilled water in a mortar until homogenized. The resulting homogenate was centrifuged at 4000× *g* for 15 min, and 1 mL of the supernatant was taken and made up to a final volume of 100 mL with distilled water in a volumetric flask.

Superoxide Dismutase (SOD) Assay: The supernatant was mixed with methamphetamine buffer (100 mM, pH 7.4), EDTA/MnCl_2_ (100 mM/50 mM, pH 7.4), 2-mercaptoethanol (10 mM), and NADH (7.5 mM). The SOD activity was determined by a spectrophotometer (Hitachi, U-2900, Tokyo, Japan) at 340 nm. In this study, one unit of SOD was defined as the enzyme activity that inhibits 50% of the NADH oxidation rate in blank samples [50].

Peroxidase (POD) Assay: The supernatant was mixed with titanium chloride (0.1% *v*/*v* dissolved in 20% (*v*/*v*) H_2_SO_4_) and centrifuged at 4000× *g* at room temperature for 30 min. The POD activity was determined by a spectrophotometer (Hitachi, U-2900, Tokyo, Japan) at 410 nm [50].

Catalase (CAT) Assay: The supernatant was mixed with enzyme reaction solution (pH 7.8, 0.98 M H_2_O_2_), the reaction solution was replaced by extraction solution, and the reaction solution was adjusted to zero. The CAT activity was determined by a spectrophotometer (Hitachi, U-2900, Tokyo, Japan) at 240 nm. In this study, one unit of CAT (U·g^−1^·min^−1^) was defined as the enzyme activity is expressed as the rate of H_2_O_2_ decline per unit mass per minute.

Malondialdehyde (MDA) Assay: The leaf samples were ground in trichloroacetic acid (TCA, 5% *w*/*v*) before being centrifuged at 10,000× *g* under 20 °C for 5 min. The supernatant was mixed with barbiturate acid (0.5% *w*/*v*, containing 20% *w*/*v* TCA) and placed in a water bath at 95 °C for 30 min before centrifuging at 3000× *g* under room temperature for 10 min. The MDA activity was determined by a spectrophotometer (Hitachi, U-2900, Tokyo, Japan) at 532 and 600 nm [50].

### 4.4. Carbon Metabolite

The carbon metabolism content of the leaves was determined using the anthrone colorimetric method [50]. Samples (0.1 g fresh rice leaves) were put into a test tube, to which 2 mL of 80% ethanol solution was added and mixed. After 3 h in a water bath at 85 °C, the supernatant was collected. This step was repeated three times, and then, distilled water was added to a volume of 10 mL. The fructose, sucrose, and soluble sugar contents were determined with the sulfuric acid anthrone method at a wavelength of 620 nm.

Fructose Content: 4 mL of 0.2% anthranone reagent was added to a test tube containing 1 mL of soluble sugar extract and mixed well. Distilled water replaced the soluble sugar extract as a blank control and was placed on ice for operation. After the reaction at room temperature for 2 h, the fructose content was determined by a spectrophotometer (Hitachi, U-2900, Tokyo, Japan) at 620 nm. The fructose content = CT × V/M, CT was the sugar concentration corresponding to 620 nm detected on the standard curve: V was the total volume of soluble sugar extract, and M was the sample weight.

Sucrose Content: 0.1 mL of 30% KOH solution was added into a test tube containing 1 mL of soluble sugar extract and mixed well. Distilled water replaced the soluble sugar extract as a blank control. After 10 min in a boiling water bath, it was cooled to room temperature. A total of 4 mL of 0.2% anthranone reagent was added and mixed well. A total of 5 mL was held in distilled water and kept at 40 °C for 20 min. The sucrose content was determined by a spectrophotometer (Hitachi, U-2900, Tokyo, Japan) at 620 nm. The sucrose content = CT × V/M, CT was the sugar concentration corresponding to 620 nm detected on the standard curve: V was the total volume of soluble sugar extract, and M was the sample weight.

Soluble Sugar Content: 4 mL of 0.2% anthranone reagent (now in use) was added to a test tube containing 1 mL of soluble sugar extract and mixed well. Distilled water replaced the soluble sugar extract as a blank control and was placed on ice for operation. After 10 min in a boiling water bath, the ice was cooled to room temperature. The soluble sugar content was determined by a spectrophotometer (Hitachi, U-2900, Tokyo, Japan) at 620 nm. The soluble sugar content = CT × V/M: CT was the sugar concentration corresponding to 620 nm detected on the standard curve, V was the total volume of soluble sugar extract, and M was the sample weight.

Starch Content: After 2 mL of distilled water was added to the residue, it was gelatinized in a water bath at 100 °C for 15 min. After cooling, 2 mL of cold perchloric acid (9.2 mol·L^−1^) was added and shaken well. After 2 mL of distilled water was added, the residue was centrifuged (6000× *g*, 5 min). A volume of 4 mL of supernatant was transferred to a 100 mL volumetric bottle. Then, 2 mL of perchloric acid (4.6 mol·L^−1^) was added into the centrifugal tube for shock. It was extracted for 15 min, 2 mL of distilled water was added, and the system was centrifuged (6000× *g*, 5 min). A volume of 4 mL of supernatant was transferred to the same volumetric bottle, and distilled water was used to hold 100 mL. The starch was determined with the sulfuric acid anthrone method at a wavelength of 620 nm.

### 4.5. Soluble Acid Invertase (SAI), Neutral Invertase (NI), Sucrose Synthase (SS), Sucrose Phosphate Synthase (SPS)

Soluble acid invertase (SAI), neutral invertase (NI), sucrose synthase (SS), and sucrose phosphate synthase (SPS) were extracted using assay kits (ZC-S0509, ZC-S0510, ZC-S0507, ZC-S0508, Shanghai ZCIBIO Technology Co., Ltd., Shanghai, China). SAI (NI) activity was measured at 480 (540) nm with the ultraviolet spectrophotometer, and the catalytic production of 1 μg reducing sugar per g tissue per minute was defined as a unit of enzyme activity. The SS and SPS activity was measured at 480 nm with the ultraviolet spectrophotometer, and the catalytic production of 1 μg sucrose per g tissue per minute was defined as a unit of enzyme activity.

### 4.6. Nitrogen Metabolite

Nitrate nitrogen: The nitrate analysis was measured by the Cataldo [51] method. A volume of 1 mL of deionized water was added to 0.1 g of fresh rice leaves. They were ground, transferred to a 2 mL centrifuge tube, placed in boiling water bath for 10 min, cooled to a constant volume, and centrifuged at 15,000× *g* for 10 min. A volume of 0.1 mL of supernatant was taken, 0.4 mL of a salicylic acid and sulfuric acid solution (5%) was added, and the system was mixed well and placed at 20 °C for 20 min. Then, 9.5 mL of NaOH solution (8%) was slowly added and cooled to room temperature. The nitrate nitrogen content was determined by a spectrophotometer (Hitachi, U-2900, Tokyo, Japan) at 410 nm.

Ammonium nitrogen: Ammonium nitrogen was measured by the Konishi [52] method. A volume of 1 mL H_2_SO_4_ (pH = 1) was added to 0.1 g of fresh rice leaves; they were ground and centrifuged at 3500× *g* for 20 min. A total of 0.1 mL of supernatant was taken, distilled water was used to fix the system to 9 mL, and 0.2 mL of sodium potassium tartrate reagent (500 g·L^−1^) and 0.3 mL of Nasi’s reagent were added, mixed, and placed at 20 °C for 30 min. The ammonium nitrogen content was determined by a spectrophotometer (Hitachi, U-2900, Tokyo, Japan) at 420 nm.

Soluble protein: Soluble protein was measured by the Ren [46] method. Samples (0.1 g fresh rice leaves) were ground up in a mortar with liquid nitrogen, to which 6 mL of a phosphate buffer (pH 7.0) was added. The extract was centrifuged at 15,000× *g* for 20 min at 4 °C, and 0.2 mL of the supernatant was combined with 9.8 mL of a coomassie brilliant blue G-250 solution (0.1 g·L^−1^). After 5 min, the soluble protein content was determined at a wavelength of 595 nm.

Free Amino Acid Content: Free amino acid was extracted using assay kits (ZC-S0653, Shanghai ZCIBIO Technology Co., Ltd., Shanghai, China), and the free amino acid content was measured at 570 nm by the ultraviolet spectrophotometer.

### 4.7. Nitrate Reductase (NR), Asparagine Synthetase (AS), Glutamate Synthetase (GOGAT), Glutamine Synthesis (GS)

Nitrate reductase (NR), asparagine synthetase (AS), glutamate synthetase (GOGAT), and glutamine synthesis (GS) were extracted using assay kits (AC-S0643, ZC-S0925, ZC-S0644 ZC-S0377, Shanghai ZCIBIO Technology Co., Ltd., Shanghai, China). The NR and GOGAT activity was measured at 340 nm by the ultraviolet spectrophotometer. Consumption of 1 µmol NADH per g fresh weight sample per hour was one unit of enzyme activity. The AS activity was measured at 420 nm by the ultraviolet spectrophotometer. Catalytic generation of glutamine to 1 nmol per g tissue per minute was defined as a unit of enzyme activity. The GS activity was measured at 540 nm by the ultraviolet spectrophotometer. A change of 0.01 per minute absorbance value per g tissue in the reaction system was defined as one unit of enzyme activity.

### 4.8. Gene Expression of Carbon and Nitrogen Metabolism

We examined the expression levels of carbon metabolism related genes (*VIN1*, *NIN1*, *SUS1* and *SPS1*) and nitrogen metabolism related genes (*AS1*, *NIA1*, *GS11* and *NADHGOGAT1*) in leaves under different light qualities. Details of the gene-specific primers and internal reference gene *OsActin3* were provided in Table 2. According to the manufacturer’s instructions, after 21 d of growth with different light qualities, the total RNA was extracted from fresh rice seedlings leaf (0.1 g) by using the RNA prep Pure Plant Kit: Polysaccharides and Polyphenolics-rich (Vazyme, China). The concentration of the RNA samples was determined using the NanoPhotometer (IMPLEN, USA). According to the manufacturer’s guidelines, the first-strand cDNA was synthesized from 2.0 μg of total RNA using the HiScript ^®^ III AII-in-one SuperMix Perfect for qPCR (Vazyme, China). The reaction volume was 10 μL, including 2 μL of cDNA, 5 μL of Hieff^®^ qPCR SYBR Green Master Mix (Vazyme, China), 0.2 μL of forward primer, 0.2 μL of reverse primer, and 2.6 μL of ddH_2_O. For the fluorescent quantitative PCR (RT-PCR, BIORAD CFX96 Touch) analysis of the gene expression, the sequences of carbon and nitrogen metabolism related genes were amplified with primers designed on the basis of the sequences obtained from NCBI (Table 2). The relative expression levels for each sample were calculated as 2^−△△Ct^ (Ct represents cycle numbers when fluorescence signal in each reaction reaches threshold). Measurement of all samples were repeated three times.

### 4.9. Statistical Analysis

Origin2018 was used to draw the cluster heat map, and Z-score was used to standardize the data in some way. A one-way analysis of variance was conducted to test the effects of different light qualities in rice early morphogenesis, carbon and nitrogen metabolism, and the antioxidant defense system, IBM SPSS Statistics 20 (IBM, Inc., Chicago, IL, USA). Different alphabetical letters are used in figures and tables for showing significant differences.

## 5. Conclusions

Combined application of red light and blue light could promote the growth of rice seedling leaves and roots under low light stress to varying degrees, increase the photosynthetic area by increasing the leaf area, improve the root characteristics by increasing the root volume, and increase the dry matter accumulation of rice seedlings. In addition, the combination of red light and blue light could increase carbon and nitrogen metabolites in rice seedling leaves, regulate the expression of genes related to carbon and nitrogen metabolism and enzyme activity, and enhance the antioxidant enzyme activity of rice seedlings. These results indicate that red light and blue light directly have synergistic effects, which can regulate the carbon and nitrogen metabolism of rice seedlings, promote the morphogenesis of rice seedlings under low light stress, and promote the growth, which have never been reported in previous studies. This study is a new discovery in the application of light quality in crop production and provides new avenues to enhance crop stress resistance. However, further study are needed to explore the physio-biochemical and molecular mechanisms of light quality in crop production.

## Figures and Tables

**Table 1:** Effects of different light qualities on morphogenesis of rice seedlings.

Variety	Treatments	Plant Height (cm)	First Leaf Sheath Length (cm)	Stem Width (mm)	Leaf Area (mm^2^)	Root Volume (mm^3^)
XZX24	W	24.36 ± 0.38 b	5.46 ± 0.11 b	0.99 ± 0.02 cd	973.09 ± 16.55 c	204.64 ± 2.02 c
	R	30.98 ± 0.21 a	6.84 ± 0.21 a	0.95 ± 0.02 d	1169.46 ± 6.57 a	186.12 ± 0.83 d
	R3B1	24.26 ± 0.36 b	5.50 ± 0.16 b	1.04 ± 0.02 c	925.41 ± 5.28 cd	268.04 ± 3.04 a
	R1B1	23.46 ± 0.70 b	5.28 ± 0.07 b	1.22 ± 0.02 b	946.09 ± 12.53 c	259.41 ± 4.68 a
	R1B3	19.26 ± 0.54 c	4.64 ± 0.04 c	1.22 ± 0.01 b	1036.45 ± 31.09 b	231.24 ± 3.14 b
	B	16.50 ± 0.25 d	4.30 ± 0.08 c	1.28 ± 0.01 a	871.43 ± 30.7 d	156.32 ± 2.55 e
HZY261	W	24.18 ± 0.84 b	5.84 ± 0.29 b	0.86 ± 0.02 bc	901.13 ± 6.83 ab	146.68 ± 1.94 c
	R	28.16 ± 0.97 a	6.74 ± 0.15 a	0.82 ± 0.01 c	937.32 ± 5.10 a	127.52 ± 3.41 d
	R3B1	23.58 ± 0.29 b	4.88 ± 0.15 c	0.84 ± 0.01 c	812.18 ± 1.77 c	210.77 ± 3.90 a
	R1B1	22.52 ± 0.82 bc	4.20 ± 0.30 d	0.92 ± 0.01 b	865.44 ± 6.77 b	195.13 ± 3.78 b
	R1B3	21.08 ± 0.38 cd	4.08 ± 0.12 d	1.04 ± 0.04 a	900.61 ± 15.23 ab	185.32 ± 6.74 b
	B	19.32 ± 0.85 d	3.92 ± 0.14 d	1.06 ± 0.02 a	867.78 ± 25.80 b	108.09 ± 0.89 e

Note: The data were presented as mean value ± standard error (SE) of three replicates. Different lowercase letters denote statistical differences between light quality treatments of a variety at the 5% level according to LSD test. XZX24: Xiangzaoxian24, HZY261: Huazheyou261, W white, R: red, R3B1: 75% red + 25% blue, R1B1: 50% red + 50% blue, R1B3: 25% red + 75% blue, B: blue.

**Table 2:** Accessions of gene and sequence of RT-PCR Primers.

Gene Name	Accession No.	Up-Primer (5′-3′)	Down-Primer (5′-3′)
*OsActin3*	Os03g0718100	CCACTATGTTCCCTGGCATT	GTACTCAGCCTTGGCAATCC
*GS11*	Os02g0735200	CACCAACAAGAGGCACAATG	ACTCCCACTGTCCTGGCAT
*NADHGOGAT1*	Os01g0681900	GTGCAGCCTGTTGCAGCATAA	CGGCATTTCACCATGCAAATC
*SUS1*	Os03g0401300	CATCTCAGGCTGAGACTCTGA	CAAATTCAATCGACCTTACTT
*SPS1*	Os01g0919400	TAGCAATGGGAAGCTGGTCT	GATCTGCTCCAGCTTGTTCC
*AS1*	Os03g0291500	TCGCAGGCGAAGAGGGCTCACGTCCTC	AGCGGGGAGACGATGGCGAGACGCTGC
*NIA1*	Os08g0468100	TTACAAGGACAACCGCGTCC	GGCGTATCCCTTCATGGTGT
*NIN1*	Os03g0314800	TGCCACTCAAGATATGCTACC	CCACAAGATTGCCAAAATAACC
*VIN1*	Os04g0535600	TGGAGCAGCAGCATACAGC	CGGATGTAAGCAGAGTTCAGC

## Data Availability

The data used to support the findings of this study are available from the corresponding author upon request.
